# ScLSTM: single-cell type detection by siamese recurrent network and hierarchical clustering

**DOI:** 10.1186/s12859-023-05494-8

**Published:** 2023-11-07

**Authors:** Hanjing Jiang, Yabing Huang, Qianpeng Li, Boyuan Feng

**Affiliations:** 1 Key Laboratory of Image Information Processing and Intelligent Control of Education Ministry of China, Institute of Artificial Intelligence, School of Artificial Intelligence and Automation, Huazhong University of Science and Technology, Wuhan, 430074 China; 2 Department of Pathology, Renmin Hospital of Wuhan University, Wuhan, 430060 China; 3 Institute of Automation, Chinese Academy of Sciences, Beijing, 100190 China

**Keywords:** Single-cell, ScRNA-seq, Siamese LSTM, Cell type detection

## Abstract

**Motivation:**

Categorizing cells into distinct types can shed light on biological tissue functions and interactions, and uncover specific mechanisms under pathological conditions. Since gene expression throughout a population of cells is averaged out by conventional sequencing techniques, it is challenging to distinguish between different cell types. The accumulation of single-cell RNA sequencing (scRNA-seq) data provides the foundation for a more precise classification of cell types. It is crucial building a high-accuracy clustering approach to categorize cell types since the imbalance of cell types and differences in the distribution of scRNA-seq data affect single-cell clustering and visualization outcomes.

**Result:**

To achieve single-cell type detection, we propose a meta-learning-based single-cell clustering model called ScLSTM. Specifically, ScLSTM transforms the single-cell type detection problem into a hierarchical classification problem based on feature extraction by the siamese long-short term memory (LSTM) network. The similarity matrix derived from the improved sigmoid kernel is mapped to the siamese LSTM feature space to analyze the differences between cells. ScLSTM demonstrated superior classification performance on 8 scRNA-seq data sets of different platforms, species, and tissues. Further quantitative analysis and visualization of the human breast cancer data set validated the superiority and capability of ScLSTM in recognizing cell types.

## Introduction

The human body comprises roughly 40 trillion cells, each exhibiting a remarkable diversity of shapes and functions [1]. Identifying and visualizing cell types offers valuable insight into cellular heterogeneity and reveals specific mechanisms underlying pathological conditions. Remarkable advancements in scRNA-seq technology have made it possible to cost-effectively and efficiently study genome-wide expression at the single-cell level. This allows us to determine cell types by examining the transcriptome status of thousands of individual cells.

Traditional sequencing methods get overall heterogeneity at the transcriptome and phenotypic levels by detecting the total signal of a cell population and calculating the average, which fails to illustrate the individual states and differences among cells [2]. The objective of scRNA-seq technology is to sequence the genome at the single-cell level, offering a distinct advantage in uncovering the subtle variations unique to individual cells and comprehending the diversity in gene expression within biological tissues. Identifying single-cell types serves as the foundation for exploring cellular heterogeneity and developmental processes.

With the ongoing innovation and improvement of scRNA-seq technology, the throughput of single-cell technology has substantially improved, enabling the detection of both common and rare cell types. scRNA-seq technologies consist not only of low-throughput sequencing technologies like Smart-Seq and Celseq but also of high-throughput sequencing technologies like inDrop, 10X-v2, and 10X-v3. The gene expression data generated by various types of scRNA-seq technologies constitute a vast and extensive database of scRNA-seq. Gene Expression Omnibus (GEO), ArrayExpress, PanglaoDB, and Human Cell Atlas (HCA), among others, are frequently used single-cell data repositories. The data used in this study were collected from the GEO database and ArrayExpress.

Among the cell type detection models based on scRNA-seq data, the models based on cell-cell similarity are the most classic. For example, Corr evaluates cell-cell associations from a global analysis of variance based on differential gene expression of cells [3]. POCR selects different kernel embedding techniques for single-cell data sets of varying sizes; Applying a Gaussian kernel for small-scale data sets and a linear kernel for large-scale data sets; On the resulting kernel similarity, spectral clustering analysis is then performed [4]. Based on the assumption that the same cell type is in the same subspace, SinNLRR describes the expression of the same cell type through a cell expression; SinNLRR finds the low-rank and non-negative representation of the expression matrix from all candidate subspaces and optimizes it using the alternating direction multiplier method (ADMM) [5]. SIMLR produces similar symmetric matrices by learning kernel functions with varying weights, then decomposes the similar matrices into approximation diagonal matrices based on the number of cell types [6]. SCENA sorts the gene expression levels in descending order of variance, selects genes to construct cell-cell similarity matrices, and combines the spectral clustering results of all matrices to obtain the final clustering results [7]. However, the above methods cannot reflect the complex nonlinear relationship between genes, affecting the cell type detection results.

Due to its robust feature learning capabilities, deep learning has proven to be advantageous when dealing with large-scale scRNA-seq data with highly complicated features. Previous research has demonstrated that neural networks can extract insights from single-cell gene expression data. The scDCC model proposed by Tian et al. encodes prior single-cell knowledge as constraint information and integrates it into the clustering procedure via a new loss function [8]. scAdapt combines domain adaptation with semi-supervised learning based on virtual adversarial training for accurate cell classification using labeled sources and unlabeled target data [9]. Song et al. proposed to use a graph convolutional network for cell type detection by constructing a mixture of inter and intra-data set cell mapping graphs [10]. The success of deep learning depends on massive datasets and robust computational resources. Despite the widespread application of deep learning frameworks such as graph neural networks and capsule networks to various problems, they continue to face the challenge of limited sample sizes, particularly in the classification of single-cell types when the amount of data varies significantly between cell types [11]. Meta-learning has been proven effective in numerous fields, such as natural language processing and robotics, due to its crucial framework for addressing the sample imbalance problem in deep learning.

Due to the varied experimental sequencing platforms in different laboratories, the distribution features of the single-cell data collected from different laboratories will vary. As indicated in Table 1, the number of samples (cells) in single-cell data sets ranges from tens to hundreds. In addition, the number of cells of various types in a single-cell data set can vary by a factor of ten. The difference in feature distribution of single-cell data sets and the imbalance of the number of cells between cell types make it challenging to identify cell types with fewer cells.

Siamese network can calculate similarity based on the distance between two samples and simultaneously enhance the learning model with the knowledge gathered from several learning events. LSTM is a unique type of recurrent neural network (RNN) that can tackle the problems of vanishing and exploding gradients during lengthy sequence training. We are interested in integrating siamese and LSTM networks to discover relevant feature representations with the potential to enhance cell type detection.

**Fig. 1:**
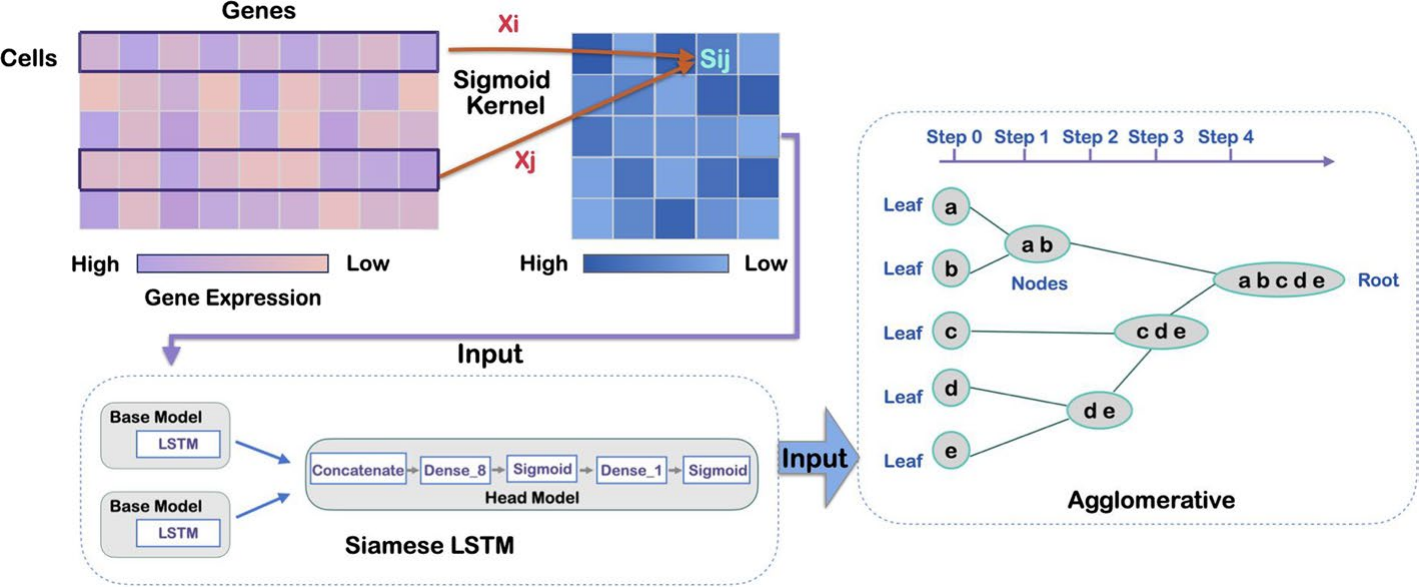
Network architecture of ScLSTM. The improved sigmoid kernel is used to build the scRNA-seq feature matrix, which is then fed into the siamese LSTM. Using the agglomerative clustering algorithm, determine the cell type of the output of the siamese LSTM

In this work, we propose a single-cell type detection model called ScLSTM, which integrates the siamese and LSTM networks. The network architecture of ScLSTM is shown in Fig. 1. ScLSTM employs an improved sigmoid kernel to calculate a measure of cell similarity. The “siamese” of a siamese LSTM is achieved by sharing weights between two identical LSTMs. Siamese LSTM takes two inputs and maps them to a new space using two LSTMs. ScLSTM learns how to minimize the distance between single-cell data of the same category and maximize the distance between different categories, enabling us to obtain more discriminative features for each cell. Subsequently, the agglomerative clustering algorithm was used to cluster single cells. The superiority of ScLSTM, compared to several state-of-the-art approaches, is validated on 8 different human and mouse scRNA-seq datasets. When comparing the three evaluation indicators, ARI, NMI, ACC, and BAS, the performance of ScLSTM in clustering is superior to that of other methods. Additionally, we apply ScLSTM to the human breast cancer dataset and two 10x Genomics datasets, demonstrating that ScLSTM can effectively categorize cancer cell subtypes. We demonstrate that the semantic representation of cells (semantic correlation between cell pairs) generated by siamese LSTM networks is more suitable for representing cells in the cell type detection problem. ScLSTM, an enhanced version of the meta-learning framework, is more effective at balancing datasets and identifying a small proportion of cell subtypes, while also performing exceptionally well on large-scale data sets.

## Methods

In single-cell-related research, the relationship between different cells can be assessed based on cell similarity calculated from gene expression. Since the scale and distribution of different single-cell transcriptome data sets are different, designing a similarity calculation method for different types of single-cell data sets is one of the significant difficulties. The Euclidean distance, Pearson, and Spearman similarity computation methods are all commonly utilized. The Euclidean distance represents the straight-line distance between two points, the Pearson correlation coefficient is used to test the linear correlation between the data, and the Spearman tests the rank correlation coefficient. We employ an improved sigmoid kernel embedding approach to express cell similarity to fully extract the global information of the single-cell data set structure and the intra-class information of the cell population.

The input of ScLSTM is the expression matrix ${\textbf{A}}$, where rows correspond to cells and columns correspond to genes. Suppose there are *n* cells in ${\textbf{A}}$, the gene expression of cell *i* is denoted by $A_i=(a_{i1},a_{i2},\dots ,a_{im})$, $i\in (1,2,\dots , n)$, where *m* represents the number of genes. Next, a logarithmic transformation is executed on each element in matrix ${\textbf{A}}$ to obtain matrix ${\textbf{L}}$, where ${\textbf{L}}=log({\textbf{A}}+1)$. For the matrix ${\textbf{L}}$, the inner product matrix ${\textbf{I}}$ is obtained by multiplying the row vectors, $I_{ij}=L_i*L_j$, where $L_i$ and $L_j$ are both row vectors of the matrix ${\textbf{L}}$. Due to the different sizes and distributions of different single-cell data sets, the inner product matrix is updated, $I_{ij} = (I_{ij}-I_{min})/(I_{max}-I_{min})$, where $I_{max}$ is the largest element in matrix ${\textbf{I}}$, and $I_{min}$ is the the smallest element in matrix ${\textbf{I}}$. Finally, the sigmoid kernel similarity matrix ${\textbf{S}}$ is calculated through the updated inner product matrix, $S_{ij}=(e^{I_{ij}}-e^{-I_{ij}})/(e^{I_{ij}}+e^{-I_{ij}})$.

### Siamese recurrent network architecture of the ScLSTM model

**Fig. 2:**
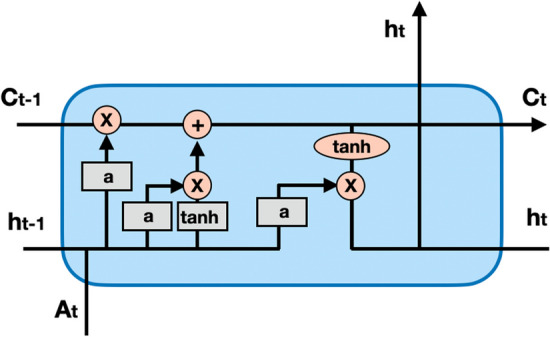
The chain structure of LSTM; Each box represents a neural network layer, consisting of weights, biases, and activation functions; Each circle represents an element-level operation; The arrow indicates the vector flow direction. A forked arrow indicates a copy of the vector

The siamese network two identical neural networks, each encoding different features of individual cells and mapping these features to a new embedding space for comparison. A siamese LSTM network is constructed by combining two identical LSTM networks with the same structure and characteristics. The LSTM structure is illustrated in Fig. 2. LSTM have two transmission states, $c_t$ (cell state) and $h_t$ (hidden state). $h_t$ can be seen as short- term memory for the current knowledge, which is transformed by the tanh function. The purpose of $c_t$ is to transform and process the memory of the last and current times using a linear transformation. LSTM is composed of three gates:

- Forget stage. The retention of past information is determined by assessing the importance of current input information. The first step in LSTM is to decide what information to discard from the cell state. This decision is made through the forget gate layer. This gate reads $h_t-1$ and $A_t$, and outputs a number between 0 and 1 for each element in the cell state $c_t-1$. The value of 1 means completely retained, while 0 means completely discarded.
- Input gate. The retention degree of input information is determined by assessing the importance of the current input information. The decision of how much new information to add to the cell state is made. Firstly, the sigmoid layer of the input gate determines which information needs to be updated. Then, a tanh layer generates a vector, denoted as $c_t$, which is an alternative for updating. These two parts are then combined to produce an update to the cell state.
- Output gate. How much the current output depends on the current memory cell. This output is based on the cell state, which is also a filtered version. First, a sigmoid layer is used to determine which part of the cell state will be output. Next, the cell state is processed by tanh (producing a value between −1 and 1), and this result is multiplied by the output of the sigmoid gate, which selects the part that determines to the output.

After performing kernel embedding, we obtain the matrix *S*. Through a base model consisting of two symmetric LSTMs, the matrix *S* is mapped to the head model. The structure of the head model is shown in Fig. 1, which consists of a concatenate layer, two dense layers, and two sigmoid layers. The output confusion matrix of the siamese LSTM serves as the input for the agglomerative clustering.

### Agglomerative clustering

Agglomerative clustering is a commonly used hierarchical clustering algorithm that can partition data sets into multiple levels to create a tree-like clustering structure. Initially, agglomerative clustering classifies each object as a cluster and subsequently merges these clusters based on a distance measure function until the desired number of clusters is achieved. In the case of ScLSTM, we apply the similarity matrix learned by the siamese LSTM to the agglomerative clustering process. Figure 1 depicts the agglomerative clustering process utilized by our method.

## Results

### Data sets and performance assessment

Human and mouse cells yielded 8 publically available scRNA-seq data sets (see Table 1 for a complete list) that were used to compare the performance of ScLSTM with other comparative techniques. Five scRNA-seq data sets were obtained from the NCBI GEO database (access IDs: GSE36552, GSE81252, GSE57249, GSE59739, and GSE81861), and one scRNA-seq data set was downloaded from the NCBI SRA database (access ID: SRP041736). Two scRNA-seq data sets were downloaded from the ArrayExpress database (access IDs: E-MTAB-3321 and E-MTAB-2600). The scRNA-seq data sets vary in size from tens to tens of thousands of cells.

Notably, the data sets we collected include those with severely unbalanced cell type,s such as GSE36552, and data sets with extremely limited data, such as GSE57249 and GSE36552. In  the GSE36552 data set, the sample size of the cell type with the largest proportion is 10 times that of the smallest proportion. There are 90 cells of 7 cell types in the GSE36552 data set and 56 cells of 4 cell types in the GSE57249 data set. Additionally, we collected a gold standard labeled data set, the Chung data set [12] (GSE75688), to further validate the robustness and generalization capability of ScLSTM. We also collected two 10x Genomics data sets to assess the performance of ScLSTM on large-scale data. The sc_10x_5cl data set (access ID: GSE118767) five cell lines (HCC827, H1975, H2228, H838, and A549) using the 10x Chromium Genomics protocol, totaling 3919 cells. The second data set is the Jurkat data set, which consist of two cell lines (293T cells and jurkat cells), using UMIs and the droplet-based protocol from 10x Genomics, with a total of 6143 cells (https://support.10xgenomics.com/single-cell-gene-expression/datasets/1.1.0/jurkat, https://support.10xgenomics.com/single-cell-gene-expression/datasets/1.1.0/293t). We applied the same preprocessing step for both 10x Genomics datasets, filtering out genes expressed in fewer  than three cells.

**Table 1:** The description of data sets used in experiments

Data set	Cells	Genes	Cell types	Protocol	Units	ID
Yan [13]	90	20,214	7	Tang	RPKM	GSE36552
Goolam [14]	124	41,480	5	SMART-Seq2	CPM	E-MTAB-3321
Camp [15]	777	19,020	7	SMARTer	FPKM	GSE81252
Pollen [16]	301	23,730	11	SMARTer	TPM	SRP041736
Biase [17]	56	25,737	4	SMARTer	FPKM	GSE57249
Usoskin [18]	622	25,334	4	STRT-Seq	RPM	GSE59739
Kolodziejczyk [19]	704	38,653	3	SMARTer	CPM	E-MTAB-2600
Li [20]	561	55,186	9	SMARTer	FPKM	GSE81861

We use three metric indicators to measure the performance of ScLSTM: normalized mutual information (NMI), accuracy (ACC), adjusted rand index (ARI), and balaced accuracy score (BAS). In particular, the ranges of NMI and ACC are between 0 and 1, whereas ARI can be negative. A larger value indicates a more remarkable agreement between predicted and actual labels. NMI, ACC, ARI, and BAS are calculated as follows:

1 $$\begin{aligned} \begin{aligned} NMI(T,P)&= \frac{2I(T,P)}{H(T)+H(P)}, \end{aligned} \end{aligned}$$

2 $$\begin{aligned} \begin{aligned} ACC&=\frac{\sum _{i=1}^{n} \delta \left( s_{i}, {\text {Map}(\text {r}_\text {i})}\right) }{n}, \end{aligned} \end{aligned}$$

3 $$\begin{aligned} \begin{aligned} \delta (x, y)&=\left\{ \begin{array}{ll}1, &{} x=y, \\ 0, &{} \text { otherwise, }\end{array}\right. \end{aligned} \end{aligned}$$

4 $$\begin{aligned} \begin{aligned} ARI(T, P)&=\frac{\sum _{ij}{n_{ij}\atopwithdelims ()2}-[\sum _{i}{a_i\atopwithdelims ()2}\sum _{j}{b_j\atopwithdelims ()2}]/{n \atopwithdelims ()2}}{\frac{1}{2}[\sum _i{a_i\atopwithdelims ()2}+\sum _j{b_i\atopwithdelims ()2}]-[\sum _i{a_i\atopwithdelims ()2}\sum _j{b_j\atopwithdelims ()2}]/{n \atopwithdelims ()2}}, \end{aligned} \end{aligned}$$

5 $$\begin{aligned} \begin{aligned} BAS&= \frac{(TPR+TNR)}{2} \end{aligned} \end{aligned}$$

where *T* represents the actual cell cluster and *P* represents the anticipated cell clusters. In equation (1), *H* denotes entropy and *I*(*T*, *P*) represents the mutual information between *T* and *P*. In equation (2), $r_{i}$ represents the predicted label, $s_{i}$ represents the true label, *n* represents the total number of samples, $Map(\cdot )$ represents the mapping function that maps the predicted labels to the equivalent true label and can be obtained by applying the Hungarian Algorithm [21]; equation (3) is the indicator function. In equation (5), *TPR* represents the probability that recall correctly predicts the positive class, while *TNR* represents the probability that the prediction for the negative class is correct.

### Influence of network structure on ScLSTM

#### Comparative analysis of clustering

**Fig. 3:**
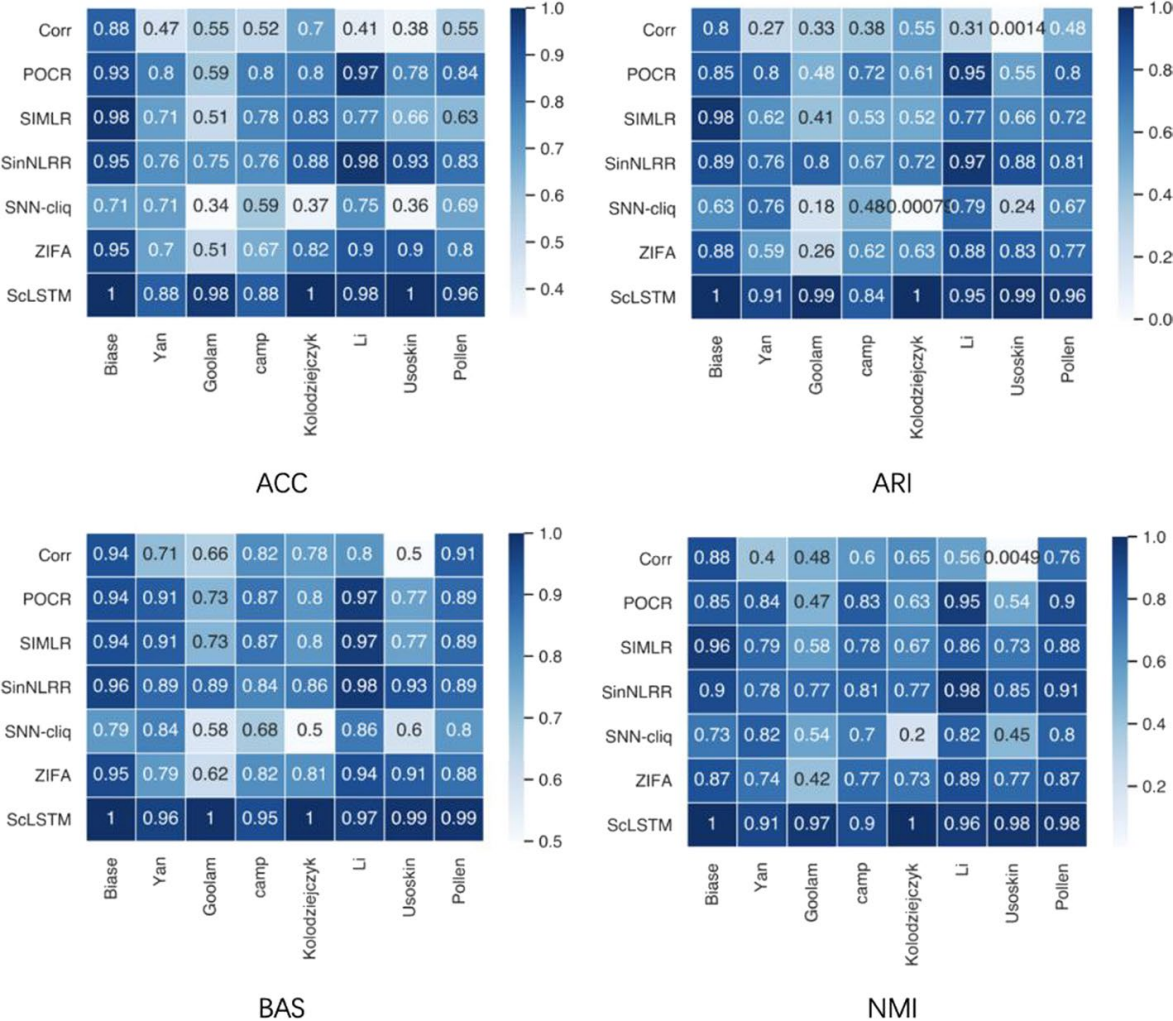
ACC, ARI, BAS, and NMI heatmaps of Corr, POCR, SIMLR, SinNLRR, SNN-cliq, ZIFA, and ScLSTM on 8 single-cell data sets

To evaluate ScLSTM, we applied it to scRNA-seq data sets generated by different sequencing platforms (see Table 1), all of which contained real labels. We choose six state-of-the-art models (Corr [3], POCR [4], SIMLR [6], SinNNLR [5], SNN-cliq [22], and ZIFA [23]) for comparison. Corr evaluates the relationship between cells based on an analysis of variance. POCR and SIMLR use kernel embedding to assess the similarity between cells. Based on similarity learning, SinNLRR imposes a non-negative low-rank structure on the similarity matrix. SNN-Cliq is a clustering algorithm based on a new shared nearest neighbor graph and quasi-clique finding techniques. Each experimental result was obtained through a grid search strategy. Figure 3 displays heatmaps of 7 models across 8 data sets using 4 evaluation metrics. Darker colors indicate closer performance to 1. The results of ScLSTM were obtained using the true number of clusters. It is evident that ScLSTM outperforms all other models in these four metrics: ACC, ARI, BAS, and NMI, followed by SinNNLR, across the 8 scRNA-seq data sets. It is worth noting that there is still a significant gap between SinNNLR and ScLSTM. The results indicate that our proposed ScLSTM performs exceptionally well on clustering tasks.

Furthermore, we compared ScLSTM with three different clustering algorithms: K-means (KM), spectral clustering (SC), and agglomerative clustering (AG). It should be noted that the overall structure of each variant algorithm still adopts the architecture of ScLSTM. Table 2 shows the results of three indicators (NMI, ACC, ARI, and BAS) on 8 data sets using three clustering methods. We can observe that agglomerative clustering almost achieves the best results, followed by K-means.

**Table 2:** The influence of KM, SC, and AG on the ScLSTM model is evaluated on eight data sets

	ARI			NMI			ACC			BAS		
	KM	SC	AG	KM	SC	AG	KM	SC	AG	KM	SC	AG
Biase	**1**	0.8825	**1**	**1**	0.9008	**1**	**1**	0.9464	**1**	**1**	0.9321	**1**
Yan	**0.9122**	0.672	**0.9122**	**0.9133**	0.8259	**0.9133**	**0.8778**	0.7111	**0.8778**	**0.965**	0.7992	**0.965**
Goolam	**0.9925**	0.604	**0.9925**	**0.9651**	0.7408	**0.9651**	**0.9758**	0.7177	**0.9758**	**0.9965**	0.782	**0.9965**
Camp	0.8197	0.6505	**0.8356**	0.888	0.8083	**0.8984**	0.8636	0.8005	**0.8841**	0.9274	0.8452	**0.9462**
Kolodziejczyk	**1**	**1**	**1**	**1**	**1**	**1**	**1**	**1**	**1**	**1**	**1**	**1**
Li	0.9641	**0.9822**	0.9527	0.963	**0.9795**	0.9573	0.9768	**0.9893**	0.975	0.9765	**0.9886**	0.9736
Usoskin	0.9771	0.9771	**0.9885**	0.9634	0.9634	**0.98**	0.9904	0.9904	**0.9952**	0.9896	0.9896	**0.994**
Pollen	**0.9584**	0.6471	**0.9584**	**0.9793**	0.8574	**0.9793**	**0.9601**	0.7143	**0.9601**	**0.9934**	0.8544	**0.9934**

The best results are shown in bold

**Fig. 4:**
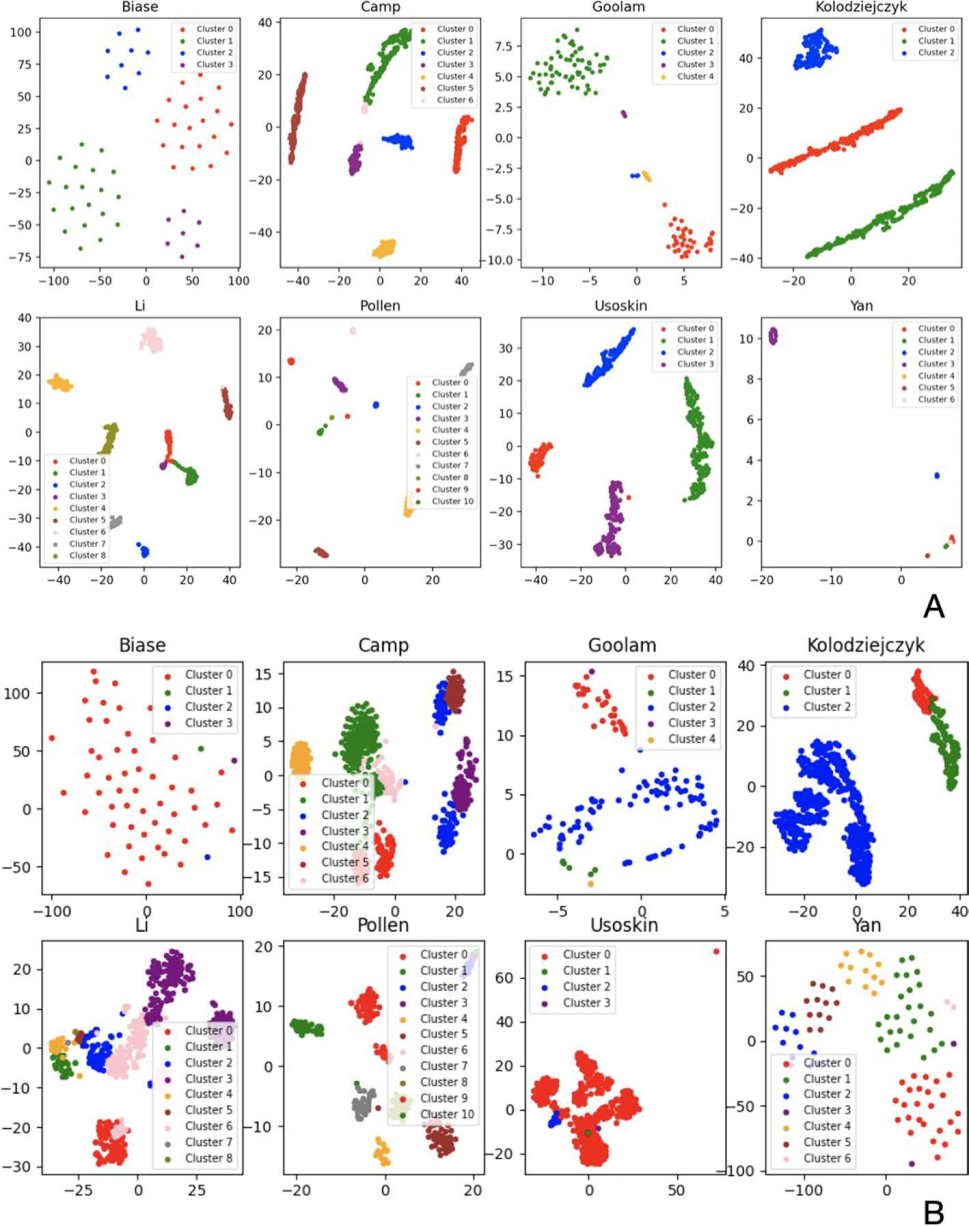
**A** The visualization results of ScLSTM on eight data sets with TSNE and annotated each cell type; **B** The visualization results of eight data sets directly after agglomerative clustering

#### Calculate the number of clusters

The number of cell types in biological research is often unknown. Therefore, determining the number of cell types within a data set is crucial for single-cell clustering method. Agglomerative clustering is implemented usingscikit learn’s function (sklearn.cluster.AgglomerativeClustering). Since POCR and SIMLR require the specification of the number of cell types, we supplemented the evaluation of the number of clusters with CORR, SNN-Cliq, and SinNLRR on eight data sets. Table 3 presents the results of four models for predicting the number of cell types across these eight data sets: ScLSTM, SNN-Cliq, SinNLR, and CORR. In addition, we used ScLSTM to predict the number of clusters to evaluate the influence of KM, SC, and AG on the ScLSTM model across eight data sets using the predicted cluster number. The experimental results are shown in Table 4.

**Table 3:** ScLTM, SNN-Cliq, SinNLRR, and CORR estimate the number of clusters on eight datasets

Data set	True clusters	ScLTM	SNN-Cliq	SinNLRR	CORR
Yan [13]	7	5	11	6	2
Goolam [14]	5	2	17	5	3
Camp [15]	7	6	32	7	4
Pollen [16]	11	10	21	11	5
Biase [17]	4	4	7	4	3
Usoskin [18]	4	5	27	4	2
Kolodziejczyk [19]	3	3	7	3	3
Li [20]	9	8	18	9	4

**Table 4:** The influence of KM, SC, and AG on the ScLSTM model is evaluated on eight data sets using the predicted cluster number

Data set	ARI	NMI	ACC	BAS
Biase	1	1	1	1
Yan	0.8965	0.8997	0.9	0.9733
Goolam	0.773	0.7527	0.8548	0.9082
Camp	0.8285	0.8926	0.888	0.9472
Kolodziejczyk	1	1	1	1
Li	0.9171	0.9358	0.934	0.9688
Usoskin	0.9865	0.9741	0.9887	0.9916
Pollen	0.9567	0.9765	0.9601	0.9932

The best results are shown in bold

#### Compared different siamese networks

To verify the effect of siamese LSTM on the effectiveness of the ScLSTM model, we propose a variant model, sigLSTM, for comparison. The sigLSTM removes one of the two identical LSTMs from the Siamese network portion of ScLSTM. The sigLSTM use the extracted feature matrix to obtain the final results through agglomerative clustering. We compare sigLSTM and ScLSTM on the 8 real scRNA-seq data sets listed in Table 1. These data sets are derived from different species and sequencing platforms, and they vary in data scale. The experimental results are presented in Table 5, which displays the optimal outcomes of ScLSTM and sigLSTM after 50 computation repeats for fourevaluation metrics (ARI, NMI, ACC, and BAS) on 8 distinct data sets. Across the Biase, Yan, Goolam, Pollen, Li, Kolodziejczyk, and Usoskin data sets, the ScLSTM model consistently outperforms the sigLSTM model. In conclusion, the ScLSTM model demonstrates superior performance across all 8 real scRNA-seq data sets generated by various single-cell sequencing platforms.

**Table 5:** The ARI, NMI, ACC, and BAS of sigLSTM and ScLSTM models are evaluated on eight data sets

	ARI		NMI		ACC		BAS	
	sigLSTM	ScLSTM	sigLSTM	ScLSTM	sigLSTM	ScLSTM	sigLSTM	ScLSTM
Biase	**1**	**1**	**1**	**1**	**1**	**1**	**1**	**1**
Yan	0.9081	**0.9122**	0.8940	**0.9133**	0.8667	**0.8778**	0.9458	**0.9650**
Goolam	0.1393	**0.9925**	0.201	**0.9651**	0.3871	0.9758	0.5665	**0.9965**
Camp	0.5399	**0.8356**	0.743	**0.8984**	0.6345	**0.8842**	0.8032	**0.9462**
Kolodziejczyk	0.0448	**1**	0.0404	**1**	0.4688	**1**	0.5229	**1**
Li	0.887	**0.9527**	0.8885	**0.9573**	0.8966	**0.975**	0.9435	**0.9736**
Usoskin	0.9801	**0.9885**	0.963	**0.98**	0.9887	**0.9952**	0.9914	**0.994**
Pollen	0.5975	**0.9584**	0.7581	**0.9793**	0.6645	**0.9601**	0.8044	**0.9934**

The best results are shown in bold

### The performance of ScLSTM on scRNA-seq data

#### Visual comparison of ScLSTM on eight datasets

**Fig. 5:**
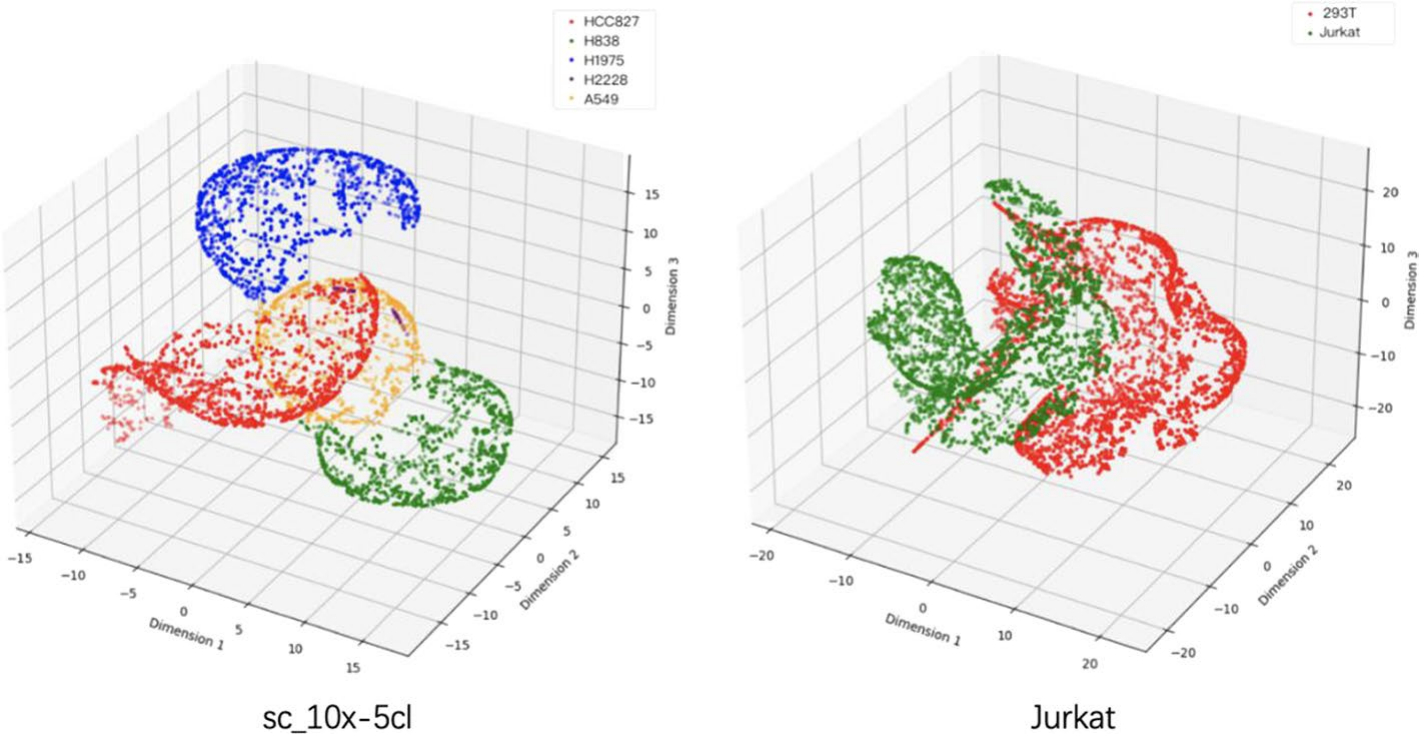
Visualization of ScLSTM results on sc_10x_5cl and Jurkat datasets

**Fig. 6:**
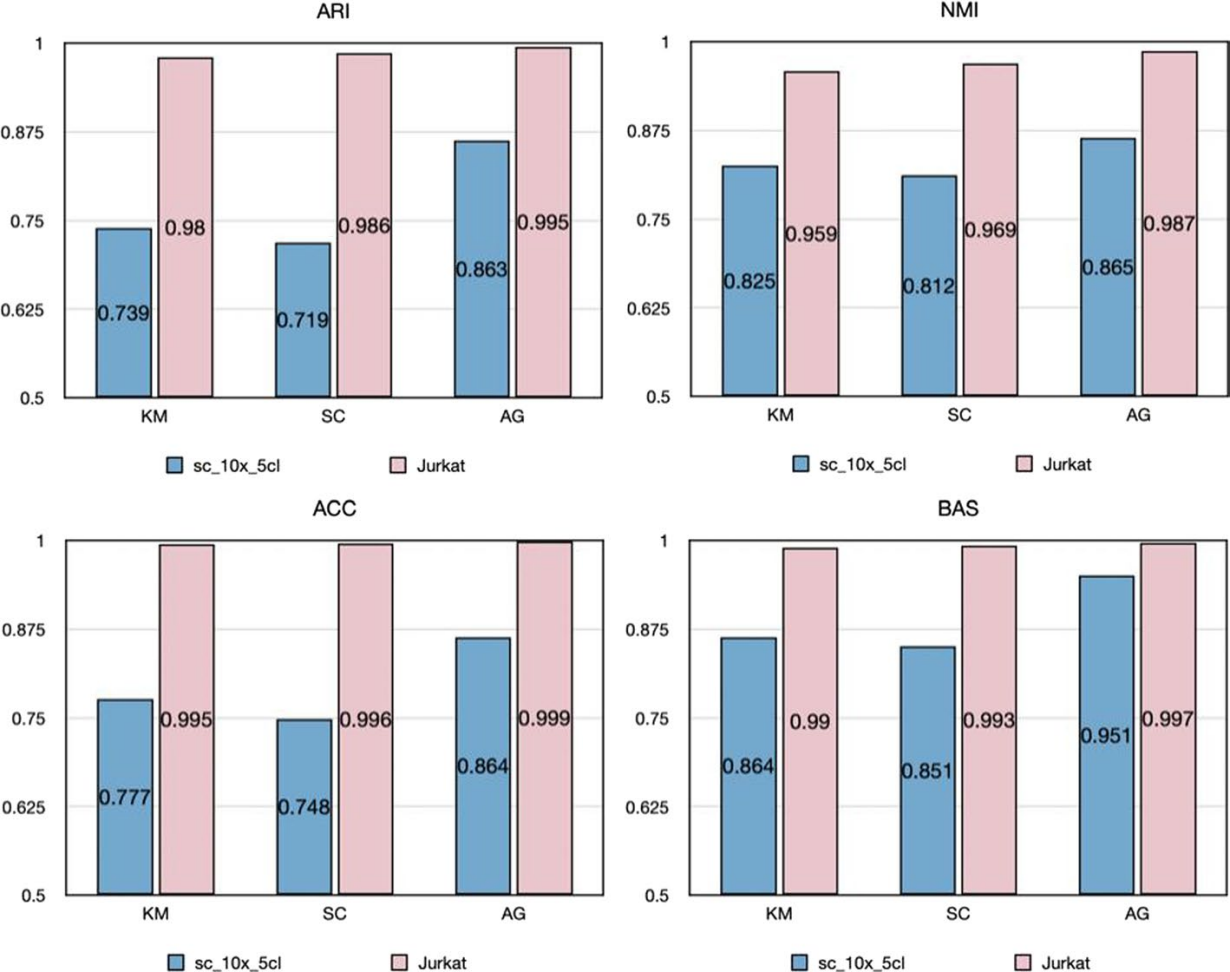
ARI, NMI, ACC and BAS results of sc_10x_5cl and Jurkat datasets based on ScLSTM model on three clustering algorithms (KM, SC, and AG)

Biologists utilize scRNA-seq data visualization techniques to identify cell subpopulations,with t-distribution Stochastic Neighbor Embedding (t-SNE) being one of the most commonly employed methods. To demonstrate ScLSTM's ability to distinguish cell types more intuitively, we visualized the results of ScLSTM on 8 data sets with t-SNE and annotated each cell type (Fig. 4A). As a comparison group, we visualized the results of 8 data sets directly after agglomerative clustering (Fig. 4B). It is evident from the visualizations that ScLSTM can effectively distinguish different cell types in each dataset, and the boundaries are clearly defined, allowing for intuitive categorization. In contrast, Fig. 4B shows that agglomerative clustering alone struggles to distinguish different categories. In the 8 data sets, various cell types are mixed to varying degrees, and not all cell categories can be distinguished in the Biase, Usoskin, and Goolam data sets.

#### Performance of ScLSTM on 10x Genomics dataset

On small-scale data sets (ranging from 50 to 800 cells), ScLSTM consistently demonstrates superior performance. To highlight ScLSTM's capability to identify cell types in large-scale single-cell RNA-seq data containing thousands of cells, we conducted experiments on two publicly available data sets obtained from the 10x Genomics website. The Jurkat data set includes both Jurkat and HEK293T cell lines. The Jurkat cell line comprises 3258 293T cells, and the HEK293T cell line includes 2885 Jurkat cells, both sequenced using UMIs and Drople-based protocols. The sc_10x_5cl dataset was sequenced using the 10x Chromium Genomics protocol. Figure 5 displays the 3D visual classification results of the two data sets after ScLSTM processing,showcasing clear boundaries between different cell categories with no confounding elements. Figure 6 presents the ARI, NMI, ACC, and BAS results of three different clustering methods on sc_10x_5cl and Jurkat data sets. It is evident that AG yields the best results. These findings demonstrate that ScLSTM performs exceptionally well on large-scale data.

### Human breast cancer cell type detection with ScLSTM

To demonstrate the robustness and generalizability of ScLSTM, we apply it to the breast cancer single-cell data set supplied by Chung et al. [12]. This dataset consists of 515 cells from 11 breast cancer patients, representing 4 types of breast cancer subtypes: luminal A, luminal B, HER2, and triple negative breast cancer (TNBC). Pathological testing confirmed the following four breast cancer markers: ER-positive (BC01 and BC02; luminal A), ER/HER2-positive (BC03; luminal B), HER2-positive (BC04, BC05, and BC06; HER2), and triple-negative (BC07-BC11; TNBC) invasive ductal carcinoma. In addition, regional lymph nodes were collected from the luminal B (BC03LN) sample and a triple-negative breast cancer (BC07LN) sample.

**Fig. 7:**
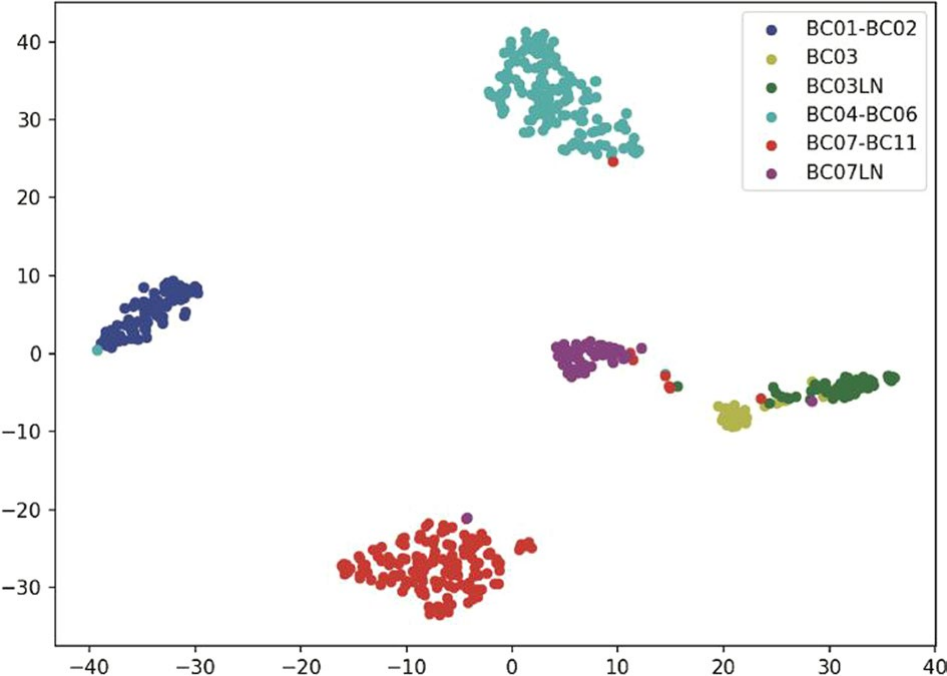
Visualization of the cells in breast cancer dataset (GSE75688) based on the t-SNE learned similarity matrix from ScLSTM

The results obtained by ScLSTM by Agglomerative Clustering are visualized using t-SNE. As depicted in Fig. 7, ScLSTM generates 6 clusters, each corresponding to a different cell type. ScLSTM separates different cell types into distinct clusters. Importantly, ScLSTM separates BC07LN and BC03LN from other cell types and maintains a clear boundary between them. It can be observed from Fig. 7 that although there are distinct  boundaries between BC03LN and BC03, the two block structures are very close, which is related to the siamese network structure of ScLSTM. Overall, all visualizations demonstrate  that the siamese LSTM structures excel at learning cell-type features and improving clustering performance.

## Discussion

In single-cell transcriptome analysis, grouping individual cells based on gene expression data is valuable for characterizing cellular composition in tissues, distinguishing developmental stages, and understanding the pathological process of tissues. Differences in the distribution of scRNA-seq data and an unequal number of cell types are significant obstacles to detecting single cell types. To address this, we propose a meta-learning model called ScLSTM, composed of an LSTM network with an improved sigmoid kernel framework. ScLSTM describes the features of each cell using various hidden units, and the cell semantic representation obtained by mining gene expression sequences is better appropriate for describing cell types.

We conducted validation experiments and in-depth analyses on 8 scRNA-seq data sets and two 10x Genomics from various single-cell sequencing platforms. Using default hyperparameters, ScLSTM consistently yields the highest ARIs, NMIs, ACCs, and BASs compared to other clustering methods. The experiment results illustrate the viability and efficacy of our proposed ScLSTM for detecting single cell types in datasets with varying scales and distributions. Unlike the Corr, POCR, and SIMLR methods, ScLSTM can directly compare the slight differences between two cells and magnify the difference between cell types.

ScLSTM’s success is contingent on its novel feature extraction and integration style. Unlike other approaches, ScLSTM locates the perspective of feature extraction at the difference between two cells, ensuring that the properties of all individual cells help distinguish between cell types. Compared to conventional single-cell type detection methods, the feature extraction from ScLSTM is more accurate and closer to the target classification. As scRNA-seq demonstrates its distinct benefits in biomedicine, we expect that ScLSTM will provide researchers with accurate single-cell clustering services.

## Data Availability

For ScLSTM, we conduct experiments on 8 different real human and mouse cells scRNA-seq data sets. The datasets generated and analysed dyring the current study are available in the Gene Expression Omnibus (GEO) repository, which were taken with URL https://www.ncbi.nlm.nih.gov/geo/. Yan, Camp, Biase, Usoskin, and Li data sets were downloaded from the NCBI GEO database (access IDs are GSE36552, GSE81252, GSE57249, GSE59739, and GSE81861), and Pollen data set was downloaded from the NCBI SRA database (access IDs is SRP041736). Goolam and Kolodziejczyk data sets were downloaded from the ArrayExpress database (access IDs are E-MTAB-3321 and E-MTAB-2600). The sc_10x_5cl data set GEO accession number is GSE118767. The Jurkat data set downloaded from https://support.10xgenomics.com/single-cell-gene-expression/datasets/1.1.0/jurkat and https://support.10xgenomics.com/single-cell-gene-expression/datasets/1.1.0/293t. The source codes of ScLSTM and the processed data sets are available at https://github.com/HanJingJiang/ScLSTM.
